# Musical Training in Congenital Hearing Impairment. Effects on Cognitive and Motor Skill in Three Children Using Hearing Aids: Pilot Test Data

**DOI:** 10.3389/fpsyg.2018.01283

**Published:** 2018-07-24

**Authors:** Sara Ghiselli, Elena Ciciriello, Giovanni Maniago, Enrico Muzzi, Sandra Pellizzoni, Eva Orzan

**Affiliations:** ^1^Department of ENT and Audiology, Institute for Maternal and Child Health IRCCS Burlo Garofolo, Trieste, Italy; ^2^Prematuri Si Nasce Association, Pordenone, Italy; ^3^Department of Life Science, University of Trieste, Trieste, Italy

**Keywords:** congenital hearing impairment, hearing aid, early intervention, cognitive ability, musical training

## Introduction

Congenital hearing impairment (CHI) is one of the most common sensory deficits (Davis and Davis, 2011) with significant repercussions on cortical brain development and behavior (e.g., Pisoni et al., 2008; Kral, 2013). Studies have shown that cortical auditory pathways develop largely on the basis of sensory experience (Kral et al., 2000; Sharma et al., 2002; Kral and Eggermont, 2007). Late treatment and rehabilitative intervention may therefore critically affect neuropsychological and language development, preventing full access to educational opportunities and hindering participation and self-reliance (Kral, 2013).

Restoration of auditory functions with current hearing device technology (i.e., digital hearing aids and cochlear implants) together with auditory training therapy are crucial to help children learn to understand how to interpret auditory signals, form meaningful sound representations, and develop their own listening strategies. Nevertheless, children with CHI still appear to lag behind their normal-hearing peers in terms of linguistic, cognitive, and motor skills (Kral and O'Donoghue, 2010).

Recent findings indicate that brain regions involved in the processing of music and language at cortical (Tillmann et al., 2006; Koelsch et al., 2009) and subcortical (Strait and Kraus, 2014) level tend to overlap, thus showing a significant degree of affinity between music and language (Patel, 2008). Music training has therefore been increasingly offered to both children with typical development (Besson et al., 2011; Kühnis et al., 2013) and children with various types of atypical development, including individuals with auditory impairments (Yucel et al., 2009; Torppa et al., 2014). Research in this field yielded the following results: (1) children with implants or hearing aids display poorer music perception skills compared to their peers (Scorpecci et al., 2012) but this seem to be related to differences in acoustical hearing prior to cochlear implantation (Hopyan et al., 2012); (2) however, when offered musical training their perception of musical acoustic features improves (e.g., Chen et al., 2010; Stabej et al., 2012; Dastgheib et al., 2013; Rochette et al., 2014; Good et al., 2017). This transfer effect of musical training seems to affect not only language (Yucel et al., 2009; Torppa et al., 2014), but also other cognitive functions (Moreno et al., 2011; Rochette et al., 2014; Benz et al., 2016). Studies mentioned so far present the following limits; (1) their main focus is on primary school children and (2) they investigate the effect of musical training on phonetic awareness, language, and memory (auditory or digit span task), disregarding all other cognitive abilities potentially influenced by music. “Even if the effect of music training on non-auditory findings are preliminary and in need of further corroboration, musical program seem to enhance working memory (Bergman Nutley et al., 2014; Zuk et al., 2014), motor and visual-spatial skills (Benz et al., 2016), and visual attention (Roden et al., 2014; Rodrigues et al., 2014).” This case report focuses on non-instrumental musical training offered to three pre-school children with CHI with the aim of investigating its effects on complex cognitive functions governing language development, such as memory, attention, and motor skills.

## Background

The study was carried out at the Audiology Unit of the Institute for Maternal and Child Health—IRCCS “Burlo Garofolo” of Trieste, Italy. Inclusion criteria for patients were set as follows: (1) CHI with mean auditory threshold between 50 and 70 dB HL, corrected with bilateral digital hearing aids; (2) age between 2 and 4 years; (3) absence of cognitive impairment; (4) permanent abode in the area of the Friuli-Venezia-Giulia region. The selection process resulted in the identification of seven eligible candidates for the study, although only three of them took part in the actual activities. The other four lived too far away from the rehabilitation center to be able to attend the training sessions. The Ethical committee of the Institute approved the project (protocol number 614/2016) and informed consent was signed by both parents of each participating child. The three participants were two girls (L.D. and S.V.I.) and one boy (N.C.). At the beginning of their musical training all three of them were aged between 35 and 44 months, had attended nursery school, and had been admitted to their first year of kindergarten. The three participants were also receiving speech-language therapy, which was not interrupted during the study.

### Measures

Individual neuropsychological evaluation was carried out before, immediately after and 6 months after the conclusion of the musical training program (MTP). Testing took place in a quite room of the hospital during 60-min sessions. In the following paragraphs each tested task is illustrated, together with the respective evaluation tool.

### Attention

Attention was measured using the Leiter-R Attention Sustained task (Roid and Miller, 1997), which ensures good internal consistency (Cronbach's α = 0.83) and good test-retest reliability (*r* = 0.85).

### Memory

Memory abilities were measured using the NEPSY tool, a neuropsychological assessment tool designed for children of 3–12 years of age. NEPSY psychometric properties have proved satisfactory (Korkman et al., 2011). The following NEPSY tasks were selected for our study:

(1) Memory for Designs: this subtest is designed to assess spatial memory by presenting new visual material. It uses a 1–20 scale (*M* = 10, *SD* = 3), with good psychometric properties as regards validity and reliability (see Korkman et al., 2011).

(2) Narrative Memory: This subtest is designed to assess recognition and (free or cued) recollection of organized verbal material. It uses a 1–20 scale (*M* = 10, *SD* = 3), with good psychometric properties as regards validity and reliability (see Korkman et al., 2011).

### Motor skills

The NEPSY task used was the Manual Motor Sequences subtest, which is designed to assess the ability to imitate a series of rhythmic movement sequences using one or both hands. It uses a 1–20 scale (*M* = 10, *SD* = 3), with good psychometric properties as regards validity and reliability (see Korkman et al., 2011).

Evaluation was integrated with two musical ability surveys, administered to the children's parents and music teacher, respectively. The two surveys are described in the following paragraphs.

#### Musical ability survey administered to the children's parents

The survey is a 26-item questionnaire; responses were rated on a Likert-type scale ranging from 1 (never) to 5 points (always). Questions concern five areas of musical ability: (a) general reaction and awareness of sounds; (b) music exposure; (c) melody and dynamic variations; (d) rhythm variations; (e) emotional response (Yucel et al., 2009). The total score was calculated as the sum of questionnaire responses over the full range of the scale, with the highest possible score set at 155 points.

#### Musical ability survey administered to the children's music teacher

The survey is a 15-item questionnaire designed by the music teacher. Responses were rated on a Likert-type scale ranging from 1 (never) to 5 points (always). Questions concern various areas of musical training (posture, synchronization, distinction between binary and ternary forms, identification of duration, reading rhythmic notation). The total score was calculated as the sum of questionnaire responses over the full range of the scale, with the highest possible score set at 75 points (Piatti, 1993; Gordon, 2003).

#### Musical training

Musical training was organized in two separate sessions with a 2-month break in-between; each session consisted of 10 individual encounters (25 min) and 10 group encounters (45 min). Encounters took place twice per week, for a total of 20 individual encounters and 20 group encounters. This training relied primarily on listening activities and did not involve the acquisition of any specific instrumental program. Activities provided a combination of motor, perceptual, and cognitive tasks, and included training in movement, rhythm, melody, sound-language production, and basic musical concepts.

In the following paragraphs each clinical case is presented individually.

### Clinical cases

#### N.C. clinical case

**Figure 1:**
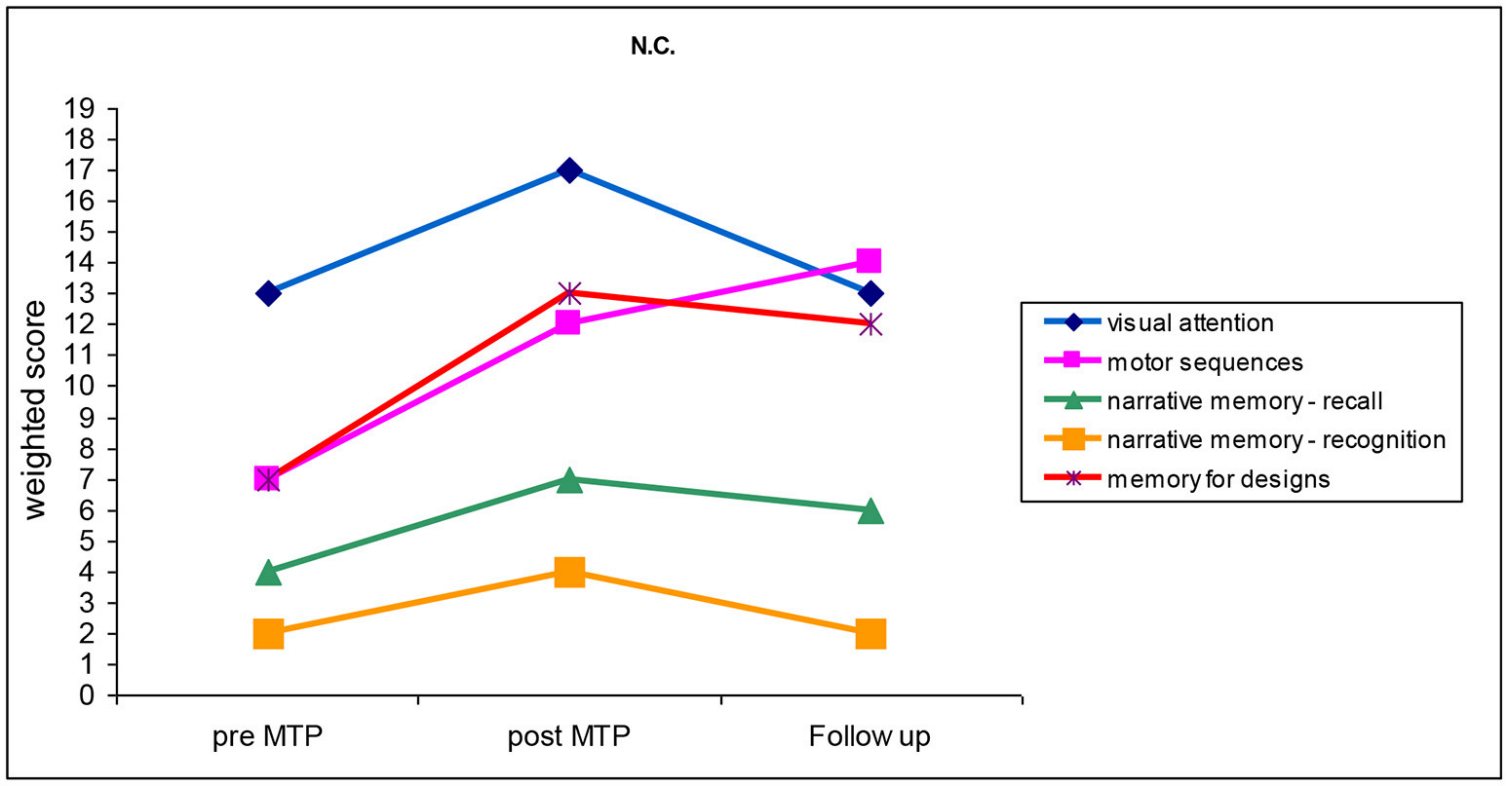
Cognitive and motor abilities performed by N.C. before the training (pre-MTP), just after the training (post-MTP) and 6 months after the training (follow-up). (*M* = 10; *SD* = 3).

N.C. was diagnosed with bilateral sensorineural CHI of moderate-to-severe degree. He had been fitted with behind-the-ear digital hearing aids since he was 3 months old. N.C. started MTP when he was 44 months old and attended 36 encounters out of 40. Before the MTP, N.C. had scored 107 in the Short Leiter QI index (Roid and Miller, 1997). Results of the neuropsychological tests administered at the beginning of the MTP (pre-MTP), immediately after (post-MTP), and during the follow-up visit (follow-up) are illustrated in Figure 1 and described in the following paragraphs.

Pre-MTP: N.C. performed very poorly in the narrative memory neuropsychological test of recognition and recall. Results obtained in the memory for drawings and motor sequences test were within the mid-lower average score. Conversely, N.C. performed well in the visual attention test, with results within the higher average bracket. Musical ability surveys returned a score of 78 points out 155 and 5 out of 75 when filled out by the parents and the music teacher, respectively.

Post-MTP: all weighted scores displayed an increase of at least two points. N.C. still performed poorly in the narrative memory test, although his post-MTP results were within the lower average score. Results obtained in the memory for drawings and motor sequences test were significantly higher in the post-MTP phase, ranking within the higher average score. N.C.'s performance in the visual attention test was above the average. Musical ability surveys returned a score of 97 points out 155 and 57 out of 75 when filled out by the parents and the music teacher, respectively, bearing further evidence of the child's improvement.

Follow up: all scores displayed a decrease, with the sole exception of results obtained in the motor coordination test. More specifically, N.C.'s performance in the narrative memory test of recognition and visual attention regressed to their pre-MTP values. Weighted scores obtained in the narrative memory test of recollection and memory for drawings test registered a slight decrease. On the contrary, N.C. obtained 133 points out of 155 in the musical ability survey administered to his parents, thus showing a consistent learning trajectory by indirect evaluation.

#### L.D. clinical case

**Figure 2:**
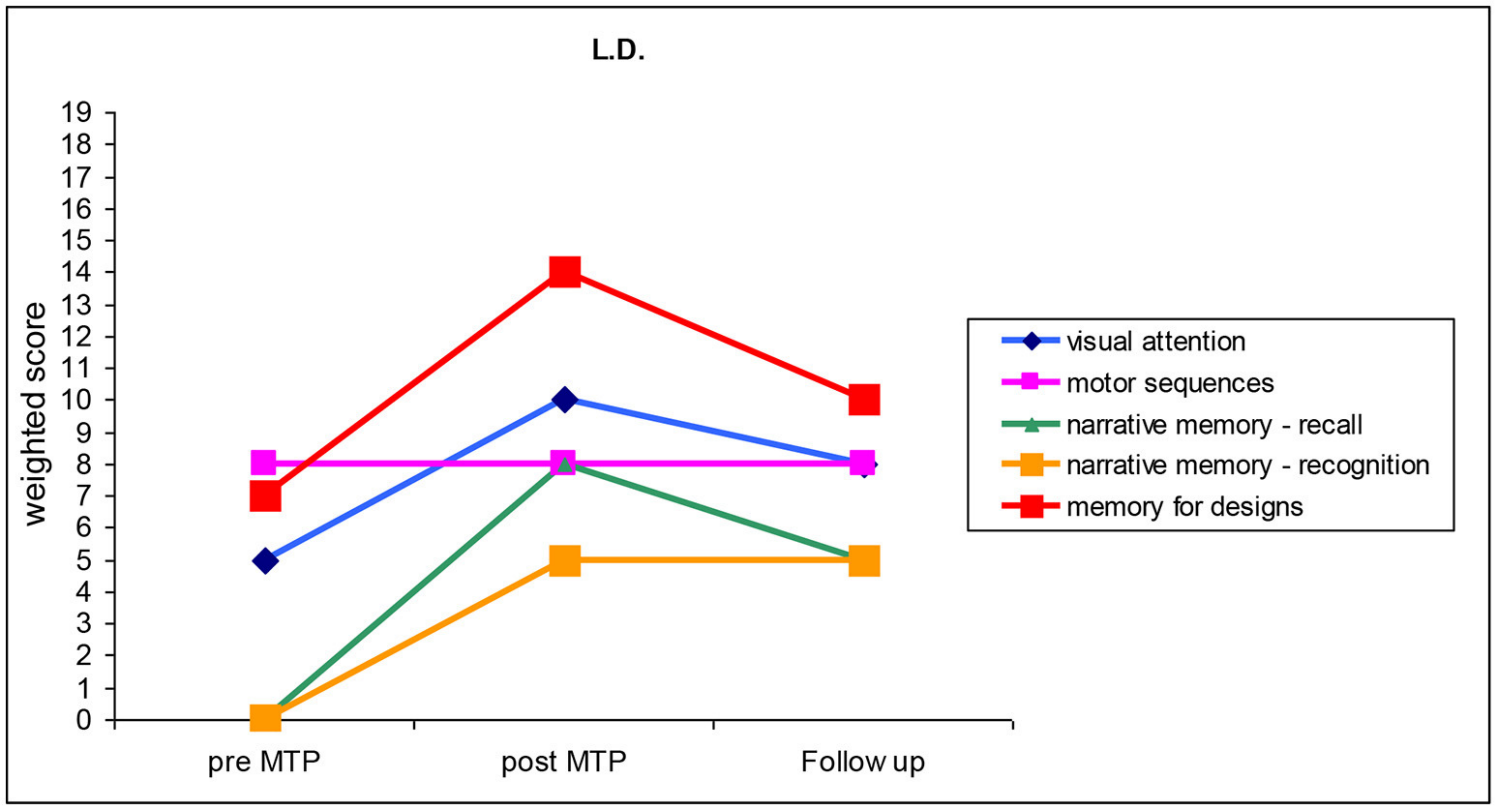
Cognitive and motor abilities performed by L.D. before the training (pre-MTP), just after the training (post-MTP) and 6 months after the training (follow-up). (*M* = 10; *SD* = 3).

L.D. was diagnosed with bilateral sensorineural CHI of severe degree. She had been wearing hearing aids since she was 3 months old. L.D. started her MTP when she was 35 months old and attended only 26 encounters out of 40. Before the MTP, L.D. had scored 105 in the Short Leiter QI index (Roid and Miller, 1997). Results of the pre- and post-MTP neuropsychological tests are illustrated in Figure 2 and described in the following paragraphs.

Pre-MTP: L.D.'s initial deficit was so severe that the narrative memory test of recognition and recollection could not be carried out. Results obtained in the memory for drawings and motor sequences test were within the average, whereas L.D.'s scores in the visual attention test could only reach the lower average score. Musical ability surveys returned a parental score of 105 points out 155 while only 3 out of 75 points in the case of the music teacher scoring.

Post-MTP: all weighted scores displayed an increase of at least five points, with the sole exception of L.D.'s results in the memory for motor sequences test. L.D. performed poorly in the narrative memory test, with post-MTP recognition scores within the lower average score. However, her post-MTP recollection results were within the average. Results obtained in the memory for motor sequences test are consistent with scores obtained in the pre-MTP phase. L.D.'s performance in the visual attention test and memory for drawings test improved, with scores ranking within the higher average score. Musical ability surveys returned a score of 150 points out 155 and 32 out of 75 when filled out by his parents and the musical teacher, respectively, bearing further evidence of the child's improvement.

Follow up: all weighted scores display a decrease, although registered values remain within the average. L.D. obtained 133 points out of 155 in the musical ability survey administered to her parents, thus showing a consistent learning trajectory.

#### S.V.I. clinical case

**Figure 3:**
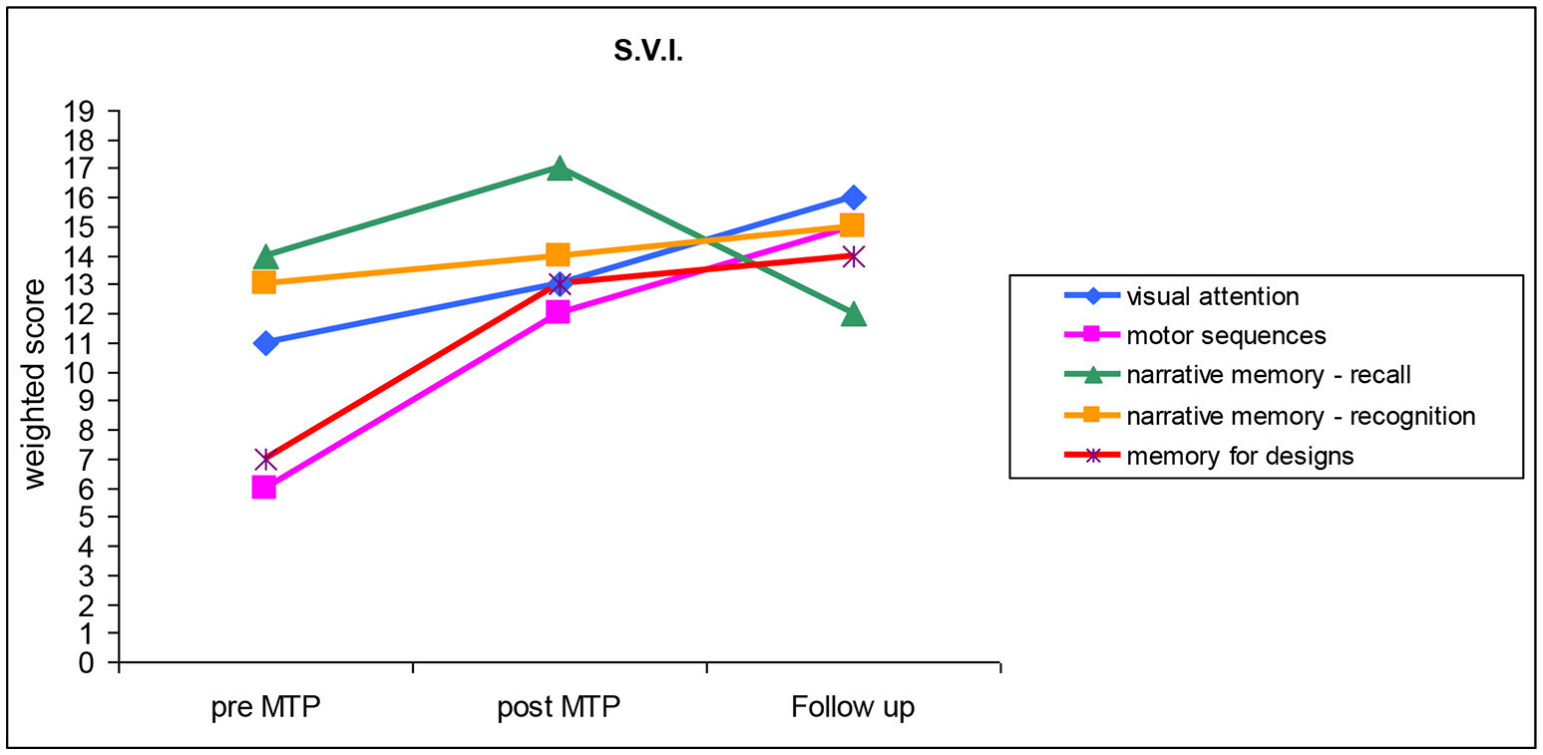
Cognitive and motor abilities performed by S.V.I. before the training (pre-MTP), just after the training (post-MTP) and 6 months after the training (follow-up). (*M* = 10; *SD* = 3).

S.V.I. was diagnosed with bilateral sensorineural CHI of moderate degree. She had been wearing hearing aids since she was 3 months old. S.V.I. started the MTP when she was 38 months old and attended 40 encounters out of 40. Before the MTP, S.V.I. had scored 123 in the Short Leiter QI index (Roid and Miller, 1997). Results of the pre- and post-MTP neuropsychological tests are illustrated in Figure 3 and described in the following paragraphs.

Pre MTP: results obtained in the memory for drawings and motor sequences test were within the mid-lower average bracket. S.V.I. obtained average scores in all the other tests. Musical ability surveys returned a parental score of 155 points out 155 and 19 out of 75 points in the case of the music teacher scoring.

Post-MTP: all weighted scores were in the mid-higher average score. S.V.I.'s performance in the memory for drawings and motor sequences test, which had obtained the lowest scores in the pre-MTP phase, registered the most significant improvement, with an increase of six points. Musical ability surveys confirmed a score of 155 points out 155 in the case of S.V.I.'s parents, while the music teacher reported an improvement with a scoring of 54 out of 75.

Follow up: S.V.I.'s weighted scores obtained in the memory for drawings and motor sequence test registered a slight increase compared to her post-M.T. values. Results obtained in the visual attention test and narrative memory test of recognition registered an improvement consistent with the scores obtained in the post-M.T. phase. S.V.I.'s performance in the narrative memory test of recollection indicates a stability in her learning process, with scores lower than those obtained in the pre-M.T. phase, although still within the higher average score. Parent again confirmed a scoring of 155 points out of 155 in the musical ability survey.

## Discussion

Our case report is an initial attempt at quantifying the beneficial effects of non-instrumental musical training on pre-school children with CHI. Tests aimed at investigating various cognitive functions, including memory, attention, and motor skills. Musical training was specifically designed to enhance children's attentional and auditory skills, focusing on the recognition of sound-related aspects, such as timbre, intensity, duration, and pitch, as well as the reproduction of rhythmic models and the combination of rhythm and melody. Investigated learning patterns also included neural-motor skills and sound-gesture coordination skills. Musical training proved beneficial in all the analyzed cognitive areas. More specifically, narrative memory, whose deficit was most significant in all three patients due to CHI repercussions on language production, registered significant improvements, with post-MTP scores ranking within the average and/or its lower scores. Previous studies indicate that patients with CHI greatly rely on their visual skills; our study confirms these results, as all three children registered a significant post-MTP improvement in the visual attention test and memory for drawings test. Such results corroborate the hypothesis according to which visual skills may serve as an effective starting point for future learning. Post-MTP results concerning memory for motor sequences were either preserved or improved, thus bearing witness of MTP's beneficial potential in this area, under-studied in the literature. Moreover, our results provide corroborating evidence of the need for promoting the development of specific motor skills in hearing-impaired children, which may result in severe motor and balance deficits (Gheysen et al., 2008) and difficulties in motor sequencing (Conway et al., 2011), when left untreated.

The report limits lay in the reduced dimension of the analyzed sample of participants, as well as in the fluctuating nature of their performance in time. We strongly believe that these preliminary data need to be further confirmed by new studies with a larger sample and with specific control groups. At the same time, however, these data seem to highlight the need for a recurrent administration of the proposed activities to ensure retention and consistent improvement (Moreno et al., 2015).

## Conclusive remarks

This case report is a preliminary observation that seems to show the efficacy of a multimodal training involving cognitive and motor skills as an effective clinical and rehabilitative tool offered to very young children with CHI using a hearing device. A combined approach may in fact enhance the child's overall skills. Further research with better methodological approach is needed to confirm the extent of benefit from specific musical training activities.

## Author contributions

SG designed the scientific work, drafted the first version of the manuscript and gave the final approval of the manuscript. EC followed the testing procedure of the children and drafted the first version of the manuscript. GM programmed and developed the musical training. EM designed and programmed the musical training. SP critically interpreted the data and gave the final approval of the manuscript. EO supervised the scientific work, provided a critical revision of the work and gave the final approval of the version to be published.

### Conflict of interest statement

The authors declare that the research was conducted in the absence of any commercial or financial relationships that could be construed as a potential conflict of interest.
